# Influence of muscle length on the stretch-shortening cycle in skinned rabbit soleus

**DOI:** 10.1038/s41598-019-54959-5

**Published:** 2019-12-04

**Authors:** Atsuki Fukutani, Tadao Isaka

**Affiliations:** Faculty of Sport and Health Science, Ritsumeikan University, 1-1-1 Noji-higashi, Kusatsu, Shiga 525-8577 Japan

**Keywords:** Bone quality and biomechanics, Neurophysiology

## Abstract

Muscle force generated during shortening is instantaneously increased after active stretch. This phenomenon is called as stretch-shortening cycle (SSC) effect. It has been suggested that residual force enhancement contributes to the SSC effect. If so, the magnitude of SSC effect should be larger in the longer muscle length condition, because the residual force enhancement is prominent in the long muscle length condition. This hypothesis was examined by performing the SSC in the short and long muscle length conditions. Skinned fibers obtained from rabbit soleus (N = 20) were used in this study. To calculate the magnitude of SSC effect, the SSC trial (isometric-eccentric-concentric-isometric) and the control trial (isometric-concentric-isometric) were conducted in the short (within the range of 2.4 to 2.7 μm) and long muscle (within the range of 3.0 to 3.3 μm). The magnitude of SSC effect was calculated as the relative increase in the mechanical work attained during the shortening phase between control and SSC trials. As a result, the magnitude of SSC effect was significantly larger in the long (176.8 ± 18.1%) than in the short muscle length condition (157.4 ± 8.5%) (p < 0.001). This result supports our hypothesis that the magnitude of SSC effect is larger in the longer muscle length condition, possibly due to the larger magnitude of residual force enhancement.

## Introduction

Muscle force generated during shortening is potentiated after active stretch. This phenomenon is called as stretch-shortening cycle (SSC) effect^1,2^. At present, the primary mechanisms are considered to be the stretch reflex^3,4^ and tendon elongation^5,6^. However, because the SSC effect was also confirmed in single skinned muscle fiber preparations which do not include the influence of neural activation and tendon elongation^7^, it is highly likely that other factors also contribute to the SSC effect. It has been suggested that residual force enhancement (RFE)^8,9^ also contributes to the SSC effect^7,10,11^ because SSCs include active stretch which induces RFE.

It is shown that RFE is, at least in part, attributable to the titin elongation, because RFE was observed at the sarcomere length of 6 μm which cannot expect cross bridge interactions^12,13^. This is in line with the results that the magnitude of RFE is prominent in the descending limb of the force-length relationship^14–16^, although the cross bridge interactions are less in the descending limb than in the plateau region. Taking this into account, it is hypothesized that the magnitude of SSC effect is larger in the descending limb because the magnitude of RFE, one of the possible mechanisms of the SSC effect, is larger in the descending limb.

Therefore, the purpose of this study was to examine the influence of muscle length on the magnitude of SSC effect. We performed the same magnitude and same velocity of active stretch and shortening within the average sarcomere length of 2.4 μm to 2.7 μm as the short muscle length condition and within the average sarcomere length of 3.0 μm to 3.3 μm as the long muscle length. We hypothesized that the magnitude of SSC effect is larger in the long muscle length condition due to the larger RFE in the long muscle length condition^14–16^.

## Methods

### Muscle samples and experimental setups

The isolated soleus obtained from New Zealand white rabbits were purchased from the SHIMIZU laboratory Supplies. The New Zealand white rabbits were euthanized according to a guideline for the Japanese Society for Laboratory Animal Resources (17-026). We adopted the soleus which is mainly composed of slow twitch fiber^17^ to minimize the influence of fatigue and/or damage. Strips of soleus muscles were harvested and tied to wooden sticks to preserve the *in situ* sarcomere length. The strips were then placed in a 50% rigor and 50% glycerol solution with protease inhibitors (cOmplete™, Roche Diagnostics, Canada) to chemically disrupt the muscle membrane. Subsequently, the strips were stored in a freezer at −20 °C for 2–4 weeks. On the day of the experiments, a single fiber of the soleus muscle was isolated using fine forceps under a dissecting microscope (SM-1TSW2-L6W-M, AmScope, US). The isolated fiber was transferred to an experimental chamber containing a relaxing solution with protease inhibitors. One end of the fiber was attached to a force transducer (Model 403A, Aurora Scientific, Canada), and the other end was attached to a length controller (Model 322C, Aurora Scientific, Canada). Sarcomere length was measured using a He-Ne laser-based diffraction system (HNLS008L-JP, THORLABS, Japan). Fiber length was measured using a microscope (SM-8TW2-144S, AmScope, US). All experiments were performed at room temperature (22–26 °C).

### Experimental procedures and measurements

**Figure 1:**
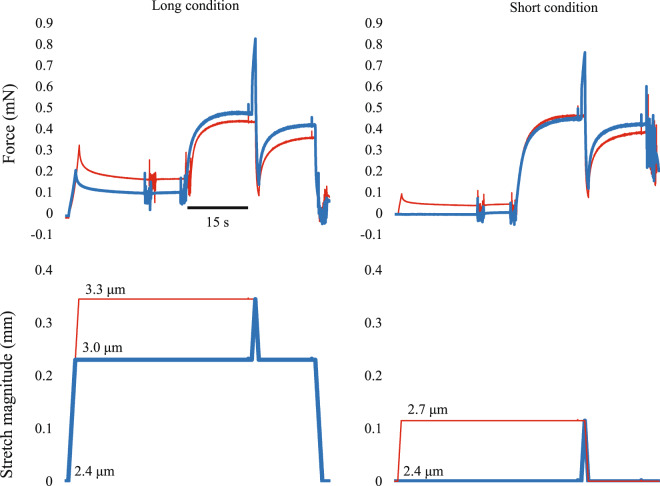
Force and length responses for the short and long muscle length conditions. Blue lines indicate the SSC trials (isometric-stretch-shortening-isometric) and red lines indicate the control trials (isometric-shortening-isometric). Average sarcomere length at a given timing is shown as the inset.

Single skinned fibers of rabbit soleus (N = 20) were subjected to short muscle length condition and long muscle length condition, and each condition included the SSC trial and control trial (Fig. 1). For the short condition, fibers were isometrically activated at an average sarcomere length of 2.4 μm and then stretched to 2.7 μm. Immediately after the end of stretch, fibers were shortened to 2.4 μm in 2 s. This is the SSC trial in the short condition. Then, the reference pure shortening contraction (control test) was performed. Fibers were isometrically activated at an average sarcomere length of 2.7 μm and then shortened to 2.4 μm in 2 s without prior active stretch. For the long condition, fibers were isometrically activated at an average sarcomere length of 3.0 μm and then stretched to 3.3 μm. Immediately after the end of stretch, fibers were shortened to 3.0 μm in 2 s. This is the SSC trial in the long condition. Then, the reference pure shortening contraction (control test) was performed after the SSC trial. Fibers were isometrically activated at an average sarcomere length of 3.3 μm and then shortened to 3.0 μm in 2 s without prior active stretch. The order of the short and long conditions was randomized. Tests were separated by a 2 min rest.

Data were collected at 10 kHz. The SSC effect was quantified by the mechanical work attained during the shortening phase. The force 15 seconds after the end of shortening was adopted for the index of RFE. The magnitude of SSC effect and RFE were expressed as the relative increase in the mechanical work or force in the SSC trials compared to that in the control trial. These values were compared between short and long conditions.

### Solutions

The recipe of solutions was the same with Fukutani *et al*.^7^ Specifically, the relaxing solution contained (in mM) 170 potassium propionate, 2.5 magnesium acetate, 20 MOPS, 5 K_2_EGTA, and 2.5 ATP, pH 7.0. The washing solution contained (in mM) 185 potassium propionate, 2.5 magnesium acetate, 20 MOPS, and 2.5 ATP, pH 7.0. The activating solution contained (in mM) 170 potassium propionate, 2.5 magnesium acetate, 10 MOPS, 2.5 ATP and free Ca2^+^ buffered with EGTA (CaEGTA and K_2_EGTA mixed in order to obtain a pCa value of 4.2), pH 7.0. One tablet of protease inhibitors was added to each 100 ml of relaxing solution.

### Statistical analysis

Descriptive data are presented as means ± SD. To examine the difference between long and short conditions, a paired t-test was used for the magnitude of SSC effect and RFE. The level of significance was set at *α* < 0.05. Statistical analyses were conducted by using the IBM SPSS Statistics version 25.

## Results

**Figure 2:**
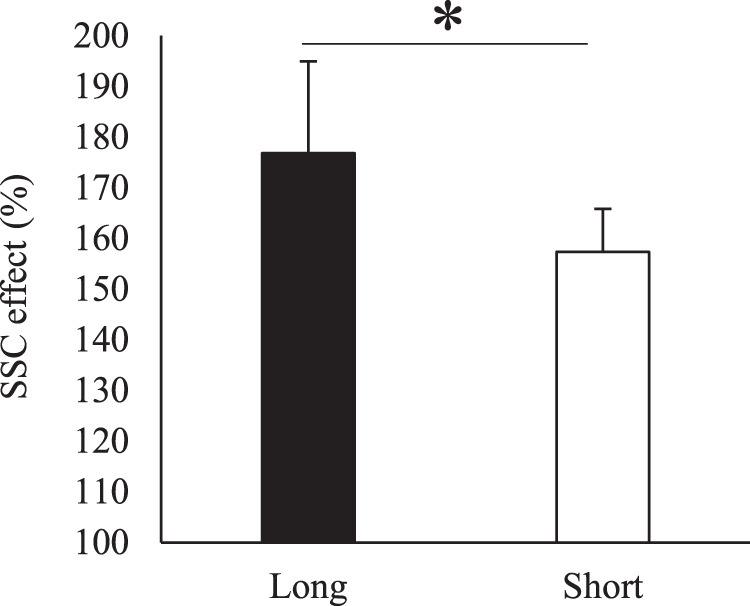
Magnitude of SSC effect for the short and long muscle length conditions. * indicates significant difference between conditions (*p* = 0.05).

**Figure 3:**
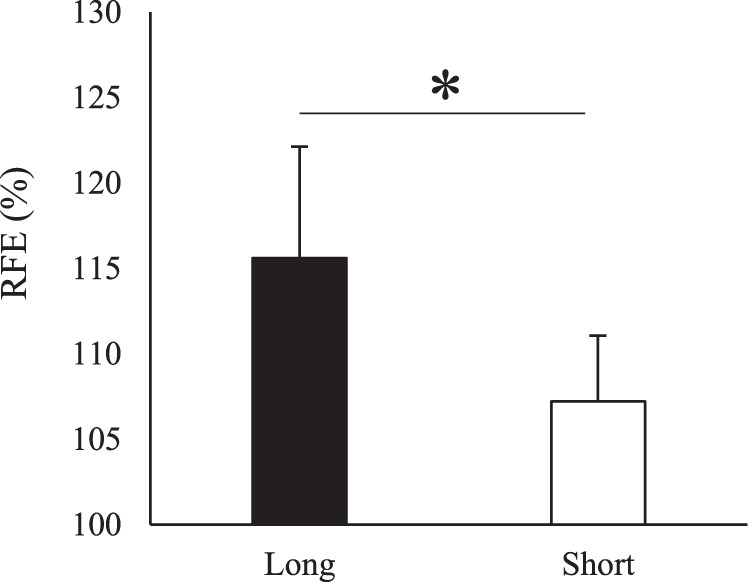
Magnitude of RFE evaluated 15 seconds after the end of shortening for the short and long muscle length conditions. *Indicates significant difference between conditions (*p* = 0.05).

The magnitude of SSC effect was significantly larger in the long (176.8 ± 18.1%) than in the short condition (157.4 ± 8.5%) (p < 0.001) (Fig. 2). Similarly, the magnitude of RFE was significantly larger in the long (115.6 ± 6.5%) than in the short condition (107.2 ± 3.9%) (p < 0.001) (Fig. 3).

## Discussion

The purpose of this study was to examine the influence of muscle length on the magnitude of SSC effect. This hypothesis was based on the fact that the magnitude of RFE is larger in the longer muscle length condition^14–16^. As expected, the magnitude of SSC effect was larger in the long condition than in the short condition. In addition, the index of RFE measured 15 s after the end of shortening was also larger in the long than in the short condition.

As hypothesized, the magnitude of SSC effect was larger in the long condition. Because we adopted single skinned muscle fiber preparations, the mechanism(s) for the observed SSC effect should be attributable to the contractile proteins in the muscle cell. The possible mechanisms are cross bridge component and titin component^7^. We observed the larger SSC effect in the long than in the short condition. The operating region for the long condition was within the average sarcomere length of 3.0 μm to 3.3 μm, while that for the short condition was within the average sarcomere length of 2.4 μm to 2.7 μm. Therefore, it is reasonable to assume that the number of attached cross-bridge was smaller in the long than in the short condition. Taking this into account, the observed larger SSC effect in the long condition should not be attributable to the cross bridge. On the other hand, the influence of titin, which is considered to be the mechanism of RFE^12,13^, is known to be greater in the descending limb than in the plateau region^14–16^. Thus, the influence of titin can explain the observed larger SSC effect in the long condition.

Regarding the contribution of RFE on the SSC effect, we have to consider the effect of shortening on the RFE because the effect of RFE should be eliminated or canceled out by shortening^18^. In fact, some studies reported no RFE after the shortening (SSC)^7,19–21^, possibly due to the above negative effects induced by shortening while others reported the existence of RFE even after shortening^11,18,22^. These contradicting results can be explained by the muscle length. As discussed, the effect of RFE is prominent in the longer than shorter muscle length condition^14–16^. Thus, one can speculate that even after shortening, the effect of RFE still exists in the longer muscle length condition. In fact, the previous study reported in the whole muscle preparations that the force at the end of shortening was enhanced only in the long muscle length condition^23^. Although they did not evaluate the isometric force after the shortening, the observed larger force at the end of shortening would be explained by RFE. To clarify this point, we measured the isometric force after the end of shortening as the index of RFE, and found that the extent of increase in the isometric force was larger in the long than in the short condition. Therefore, it is reasonable to assume that the magnitude of RFE was larger in the longer muscle length condition. This result strengthens the hypothesis that whether the effect of RFE exists after the end of shortening is, at least in part, explained by the operating region of muscle (muscle length). Based on this finding, the effect of SSC derived from RFE is expected to occur when muscles behave under the longer length, indicating that the effect of SSC is motion-dependent in the case of human movements.

In conclusion, the SSC effect is larger in the longer muscle length condition. This may be caused by the contribution of RFE which is prominent in the longer muscle length conditions (descending limb).
